# Healthcare access for autistic adults: A systematic review

**DOI:** 10.1097/MD.0000000000020899

**Published:** 2020-07-17

**Authors:** Shenae Calleja, Fakir M. Amirul Islam, Jonathan Kingsley, Rachael McDonald

**Affiliations:** Faculty of Health, Arts and Design, Swinburne University of Technology, Hawthorn.

**Keywords:** adults, autism spectrum disorder, barriers, enablers, healthcare access

## Abstract

**Background:**

People with autism spectrum disorder (ASD) have an increased susceptibility for many chronic health conditions compared with their peers. An increasing number of adolescents are transitioning from pediatric to adult healthcare services. Thus, being able to access appropriate healthcare services that can not only address specific needs of the person but enable them to better manage healthcare conditions and decrease the development of preventable disease is necessary. A systematic review was conducted to identify barriers and enablers of healthcare access for autistic adults.

**Methods:**

The studies included in the review were quantitative and qualitative and were published between 2003 and 2019. The participants for the review are considered to be adults (over 18 years of age) with a primary diagnosis of ASD.

**Results:**

In total, 1290 studies were initially identified and 13 studies were included based on the inclusion and exclusion criteria outlined in a previous protocol paper. The analysis of these studies identified areas of concern to access appropriate healthcare, such as clinician knowledge, the environment, and life events.

**Conclusion:**

Identifying the barriers to healthcare, highlights ways healthcare services can regulate scope of practice, the physical environment, and the process of managing health conditions, thus, autistic adults can strive for optimal health. This review contributes to peer-reviewed evidence for future research and up-to-date information when developing and piloting health interventions for autistic adults.

**Ethics and dissemination:**

There are no human participants, data, or tissue being directly studied for the purposes of the review; therefore, ethics approval and consent to participate is not applicable.

**Registration and Status:**

PROSPERO 2018 CRD42018116093

## Abstract 2

Supplemental Digital Content is available in the text

## 1 Introduction

Autism spectrum disorder (ASD) is a neurodevelopmental condition where people may experience difficulties in social communication and social interaction skills, restricted interests, and repetitive behaviors.^[1–4]^ ASD can impact a person and their supports throughout the lifespan.^[5]^ Autistic people have an increased susceptibility to physical, mental, and social health issues.^[4,6,7]^ The prevalence of ASD is estimated to be 1 in 160 children worldwide.^[8]^ The implications of this increase in diagnosis is that there will be a substantial number of autistic adults transitioning from the pediatric healthcare system to the adult healthcare system. At the same time, people with ASD have been shown to experience health disparity compared with their peers.^[9]^

Autistic people are frequently reported to experience more common chronic health conditions than their peers, such as seizure disorders (11.9% compared with 0.73%), hypertension (25.6% vs 15.6%), and allergies.^[4]^ Mental health difficulties are also common, such as anxiety, bipolar disorder, dementia, depression, and schizophrenic disorder.^[4,6,10–12]^ Autistic people have also been reported to frequently demonstrate behaviors of concern such as aggression, property destruction, disruptive, and self-injurious behavior,^[13–16]^ which may further interrupt their ability to access appropriate healthcare. Most notably, numerous studies have indicated that many common chronic health conditions, were significantly more common in autistic adults than their non-autistic peers.^[6,7]^ Multiple life factors can impact the overall health of an autistic person^[17,18]^ and consequently create barriers to access appropriate healthcare. These multiple factors can include, the ASD diagnosis, the age of diagnosis, living situation, level of education, employment opportunities, or family and friend support.^[17,18]^

Most of ASD research concentrates on children and adolescents,^[5,19,20]^ and there are several reviews on disparities in healthcare for pediatrics, barriers for vaccinations, and autism intervention.^[21–24]^ The health disparities experienced by autistic adults, including barriers and enablers to healthcare remains unexplored, not quantified, and remains a question. That is why we are undertaking this project and the systematic review.

A review conducted by Tregnago and Cheak-Zamora^[25]^ considered disparities in healthcare, in a pediatric population. More recently, a systematic review by Mason et al^[26]^ explored physical healthcare services for autistic adults, which only included 6 studies. One of the reasons for this was the included studies focused on the views of autistic people.^[26]^ To date, there is no systematic review evidence on overall healthcare access for autistic adults from multiple perspectives and the level of healthcare where barriers and enablers are present. Given the increased prevalence of chronic health conditions and the incidence of preventable health problems experienced by autistic adults, ensuring this population has access to appropriate healthcare is vital, and a review that aims to identify known barriers and enablers may assist when developing and implementing appropriate health interventions in the future.

Reviewing the evidence for healthcare access for autistic adults will identify ways healthcare clinicians can regulate scope of practice, the environment and appropriate management of health conditions, thus, autistic adults attain optimal health. This systematic review will explore healthcare access, and appropriate services for autistic adults. An up-to-date systematic review is imperative for clinical practice^[27]^ and the results will provide peer-reviewed evidence for future research directions when developing and piloting health interventions for autistic adults. The primary question is what are the barriers and enablers of healthcare access for adults with ASD; and how can healthcare access for adults living with ASD be enhanced? Our hypothesis was that poor communication plus a lack of understanding of autistic needs are the main barriers to access appropriate healthcare in primary settings and the literature exploring this topic is sparse.

## 2 Methods

### 2.1 Search strategy

This review is registered on the PROSPERO database (CRD42018116093) and the protocol is published.^[28]^ A university librarian with experience in systematic reviews assisted with the database search strategies. Databases searched were EBSCOhost, Scopus, PubMed, The Cochrane Library, and Web of Science applying this search strategy and the journal *Autism* was manually searched. A sample of a search strategy used to extract relevant articles was ((Autism Spectrum Disorder OR Autism OR ASD OR Neurodevelopmental Disorder OR Asperger's OR Pervasive Developmental Disorder) AND (Healthcare OR Health Services OR Health Care OR Health Management OR Hospital OR Medical OR Health Maintenance) AND (Barrier OR Boundary OR Challenge) AND (Enable∗ OR Facilitat∗)). The full search strategy is included in the supplementary material (Supplementary Material 1). Searches were conducted between November 2018 and July 2019 for any eligible recent published articles.

### 2.2 Inclusion and exclusion criteria

One reviewer (SC) independently screened data from eligible articles and resolved any differences by discussion. All authors reviewed the titles and the results and compared with the eligibility criteria, to increase validity (RM, AI, JK). The EndNote program^[29]^ was employed as a database and utilized for the screening of articles. The inclusion criteria were as follows: a primary diagnosis of ASD although intellectual disability is a co-occurring condition^[6]^ and was included as a co-condition. Study types included were original peer-review research articles with a date from 2003 to 2019—a 16-year range. The participants for our review were considered to be adults (over 18 years of age) with a primary diagnosis of ASD. The search was limited to English and limited to adults living with ASD. No unpublished data were included. Our exclusion criteria were pediatric studies—children under the age of 18, ASD not the primary diagnosis, studies of parents of children under the age of 18, review papers (systematic or narrative), book chapters, commentary articles, opinions, letters, and editorials. The primary outcome was to identify barriers and enablers of healthcare access for autistic adults. A further level of analysis was conducted to identify the level of healthcare that needs further support to better access healthcare services for autistic adults.^[28]^

### 2.3 Quality assessment, data extraction, and synthesis

The quality of studies were assessed using the Mixed Methods Appraisal Tool (MMAT).^[30]^ The data were extracted using a data extraction form which included the studies for assessment of quality and evidence synthesis (Supplementary Material 2).

Extracted information included quantitative studies: number of participants; source of participants (primary healthcare, secondary healthcare, tertiary healthcare); participant demographics (age, sex); geographic location; type of study; other co-conditions included; reported outcomes and statistical significance, and level of healthcare identified that may need further support.

Qualitative studies: number of participants; source of participants (primary healthcare, secondary healthcare, tertiary healthcare); participant demographics (age, sex); geographic location; type of study; other co-conditions included; category- or theme-level evidence from the findings or results section of the included papers, and level of healthcare identified that may need further support.

All quality assessment and data extraction were carried out by 1 reviewer and checked in detail by all reviewers. We completed a narrative synthesis of the results. A narrative synthesis is a systematic approach for undertaking a review where statistical methods or pooling of the data cannot be performed.^[31]^ Narrative synthesis is an approach that will enable the investigation of similarities and differences and highlight the quality of published evidence to inform practice or policy.^[31]^ We organized the studies inductively into broad categories of study design and summarized each study using the data extraction form for the study characteristics. Calleja et al^[28]^ proposed to use the Risk of Bias tool (RoB 2.0)^[32]^ although during the analysis, it was not applicable for the included studies.

## 3 Results

**Figure 1:**
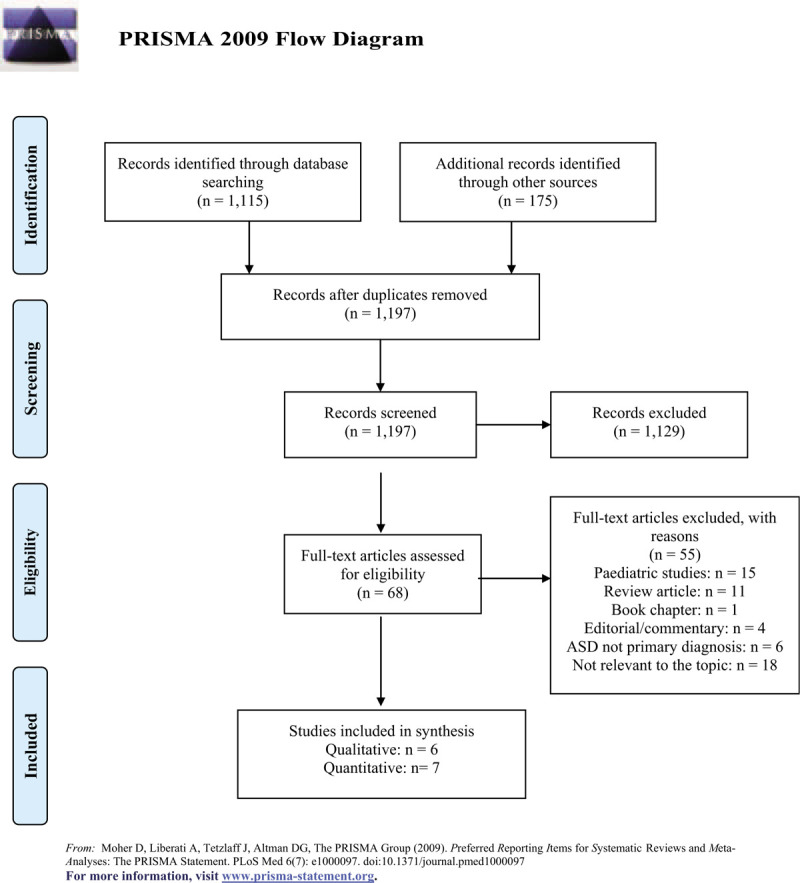
Prisma flow diagram.

A total of 1290 articles were identified through the original search process, of which 93 were duplicates. Based upon title and abstract screening, 1129 articles were excluded as they did not meet inclusion criteria. Sixty eight articles were determined to be eligible and a full-text review was completed. The remaining 13 studies were included in the review. The screening process is detailed in Fig. 1—flow diagram.^[33]^

Table 1  summarizes the evidence examining barriers and enablers of healthcare for autistic adults. In the following synthesis, we characterize the evidence, describe each study's findings, present a summary of each category, and determine the level of healthcare that may need further support. Of the selected articles, 7 used qualitative synthesis, 5 used quantitative synthesis, and 1 was a mixed-methods synthesis. Participants included in the studies were adults with ASD (N = 9), parents/families (N = 2), and healthcare providers (N = 2). Most studies were carried out in the United States (N = 5), followed by the United Kingdom (N = 3). In all, 7 studies used a qualitative design, 2 used a non-randomized controlled design, 3 used a quantitative descriptive design, and 1 study used both a non-randomized controlled and quantitative descriptive design. The majority of studies highlighted that primary healthcare needed further support for autistic adults (N = 10).

**Table 1:**
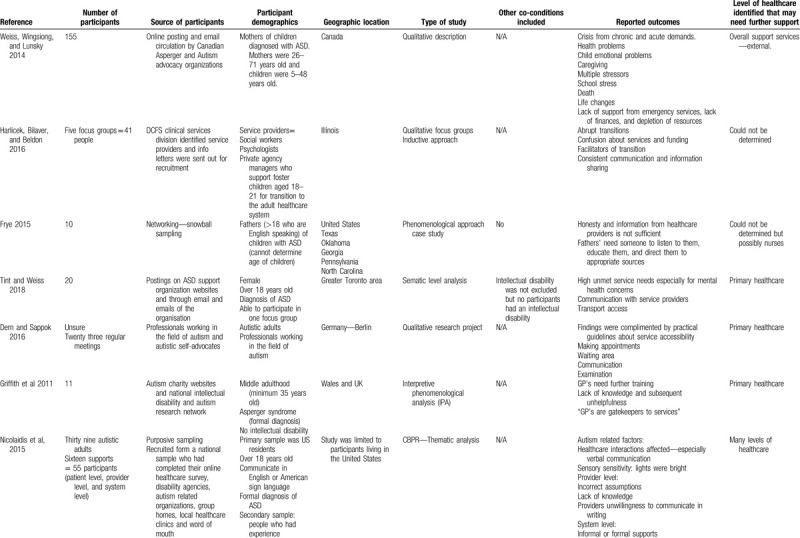
Summary of evidence examining barriers and enablers of healthcare for autistic adults.

**Table 1 (Continued):**
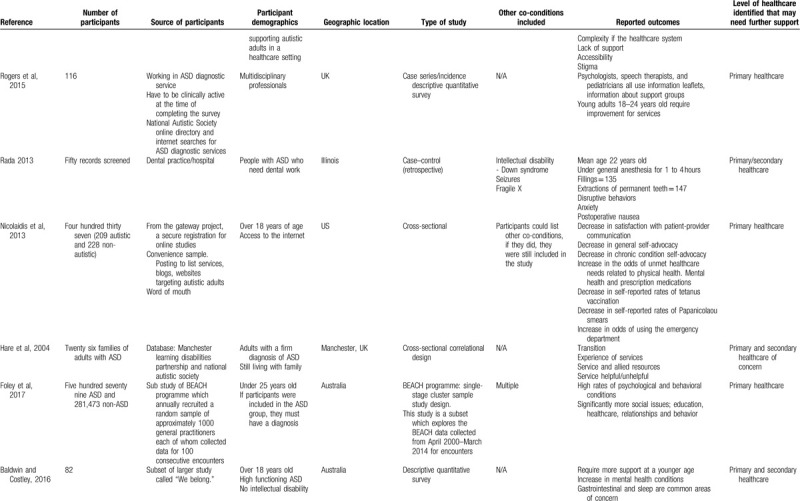
Summary of evidence examining barriers and enablers of healthcare for autistic adults.

There was a variance in opinions for the type of studies, the outcomes and the quality of evidence, however, all authors came to a consensus using the MMAT (Supplementary Material 3). The studies were divided into the following 2 categories of identified barriers to healthcare: Barriers to healthcare included: health conditions, communication, and life changes. Barriers to organizational healthcare provision included: support from health practitioners, lack of knowledge about ASD, care coordination, and environmental factors. A total of 8 studies evaluated autistic adults and the experiences of healthcare, a total of 2 studies evaluated the health needs of autistic adults, and 3 studies evaluated other outcomes regarding healthcare and autistic adults.

### 3.1 Barriers to healthcare consisted of 3 areas

#### 3.1.1 The type of health conditions experienced by autistic adults compared with the general population:

One of the barriers to accessing appropriate healthcare could be due to the type of health problems autistic adults are likely to experience,^[34]^ which also impacts family caregivers. Health problems vary from chronic and physical health conditions^[35]^ to mental health conditions.^[35–39]^ Nicolaidis et al^[35]^ identified that autistic adults had higher physical health needs (odds ratio [OR] 1.9 confidence interval [CI] 1.1–3.4) compared with non-autistic people and higher mental healthcare needs (OR 2.2, CI 1.3–3.7) compared with non-autistic people.

#### 3.1.2 The ability of the autistic person to communicate their health needs

Poor miscommunication may be due to multiple factors, some factors identified by Tint and Weiss^[37]^ is focused on the language used from the practitioner and inexperienced experts. This suggests that the communication mode may have to be addressed for transparency and to build strong provider–patient relationships to adequately address healthcare issues and concerns.

#### 3.1.3 Life changes

While all people experience life changes, for people with ASD families and caregivers may be the focus of healthcare intervention rather than the person themselves. Further, most people transitioning from childhood to adulthood do not require a handover of services; yet for this population, going from a well-supported pediatric system to a fragmented system with an emphasis on self-management is difficult. A total of 3 studies highlighted that life changes may impact the lives of people living with ASD.^[34,40,41]^ Hare et al^[40]^ and Weiss et al^[34]^ focused on family carers and crisis from acute demands, such as caregiving, multiple stressors, and lack of support from healthcare providers. Hare et al^[40]^ and Havlicek et al^[41]^ identified a significant importance in the transition period for people with ASD from the pediatric to adult healthcare system. There is limited coordination, the transition is usually fast which can be distressing and there is a lack of attention to personal needs.^[41]^

### 3.2 The organization of healthcare provision is often a barrier due to a number of factors. These include

#### 3.2.1 Support from health practitioners and clinician dishonesty

Frye^[42]^ and Weiss et al^[34]^ identified that there is a lack of support from service providers, particularly, from general practitioners and the hospital system. Moreover, dishonesty and lack of information from healthcare providers were also reported as barriers in the healthcare system.^[42]^ The participants in this study were fathers of autistic people and they expressed that the best information would come from people that have similar experiences.^[42]^

#### 3.2.2 Lack of knowledge of autism

Nicolaidis et al^[43]^ identified a number of factors impacting the personal level, provider, and system level. Healthcare interactions are important, they can be affected if verbal communication is unclear.^[44]^ The outcomes for the provider level barriers were incorrect assumptions, lack of knowledge, and providers unwillingness to communicate in writing. Four studies addressed communication, between the provider and patient.^[35,37,43,45]^ The system level barriers were highlighted around the supports for autistic people, the complexity of the healthcare system, accessibility and stigma.^[44]^ This suggests that many levels of support can impact healthcare access for autistic adults.

#### 3.2.3 Coordination of care

Healthcare providers play a vital role in access and delivery of healthcare for autistic adults. One study noted that general practitioners are fundamental in the coordination of health as they are the gatekeepers to services.^[46]^ Three studies highlighted the lack of knowledge from health service providers.^[43,46,47]^ One study highlighted that incorrect assumptions led to stigma about ASD^[43]^ and in turn suggests that these system level factors that impact healthcare can impact an individual's wellbeing.

#### 3.2.4 Environmental factors and sensory sensitivity

One study focused on the environment playing a role in the access to healthcare for autistic adults.^[45]^ There may be difficulties making phone calls, the physical environment of the waiting area may make a person feel anxious, the stress of the uncertainty (time), physical closeness to other people, sensory overstimulation, and disturbing sounds. The sensory sensitivity may also be a barrier to accessing health as the environment may make a person feel safe or unsafe, for example, the lights may be too bright.^[44]^

## 4 Discussion

Accessing appropriate healthcare to reduce health problems is part of the international health agenda for autism, however, autistic people have poorer health compared with non-autistic people. This systematic literature review examined the known healthcare access barriers faced by autistic adults. The study included quantitative and qualitative study designs. Firstly, it is imperative to identify and understand the different barriers and enablers autistic people experience as ASD is a complex lifelong condition, which can have a potentially detrimental impact on adult functioning.^[9]^ Secondly, examining healthcare access barriers and enablers assists in identifying pathways for future pilot interventions that contribute to healthcare for autistic adults in providing opportunities to better improve healthcare access by developing appropriate resources/tools. Finally, clarifying the current literature focusing on autistic adults and healthcare access is important for streamlining and directing further research efforts for future interventions. The level of healthcare that needs further support is important to identify as future research efforts can develop interventions to support practice.

Autistic adults are more likely to have multiple health conditions compared with the general population and the type of healthcare accessed can have detrimental impacts on adult functioning.^[34]^ The evidence on barriers and enablers for autistic adults is limited, and the available evidence suffers from reliability. Most studies have a small sample size that impacts in drawing any appropriate conclusions. Moreover, most of the studies have primarily been conducted in the United States and the United Kingdom, and no studies measured the same variables. One study focused on the outcomes of the barriers for autistic adults and the delivery of healthcare, but the authors combined 2 studies in 1, the discussion was based on the validation of a tool, but identified the population and used 2 designs, the title implies comparisons of healthcare for autistic and non-autistic people, but the discussion of the study was specifically about validating a tool.^[35]^ One of the barriers is specifically due to miscommunication between autistic adults and healthcare providers. Appropriate communication between the provider and the patient is essential, although, some clinicians may not describe the health result or future referrals in a way that the autistic adult may understand.^[35,43]^ Many autistic adults receive healthcare for their mental health, and this is well supported.^[10,35,37–39]^ Regarding overall wellbeing, health clinicians need further support around the types of services autistic adults can access, health services that are local, and health services that are relevant to individual needs.

The evidence for life changes such as the confusion of accessibility of future services for autistic people is very limited compared with healthcare in general.^[41]^ Transitioning from the pediatric to the adult healthcare system can be overwhelming and difficult to locate relevant and appropriate services for autistic people. This study focused on foster youth transitioning to the adult healthcare system, however, the change of clinicians can also be of concern, where the history of the patient will have to be understood. These changes are important and vital for wellbeing and care of all autistic adults, from our review, interventions for autistic adults around the transition period from the pediatric to adult healthcare system is limited and this can be problematic in the future.^[41]^ An existing study by Nicolaidis et al^[43]^ identified recommendations specifically for online toolkits that clinicians can access to increase knowledge and confidence for their patients with autism. This toolkit enables clinicians to better support autistic patients, however, clinicians need to be informed of such toolkits that are available for use. The toolkit provides an understanding of autism, the diagnosis process, and relevant referrals to specific disciplines. A synthesis of the studies reveals that the level of healthcare that needs further support is primary healthcare. This finding has various implications to the access and delivery of healthcare. Since, general practitioners are the first point of referral, clinicians need to understand what is autism and what support an individual and their family may need.^[10,35,37–40,43,45–47]^ Likewise, with the understanding of what services an individual will need to access, the physical environment needs to be considered for each practice.^[45]^ The lighting, sound, and activities need to be reviewed and considered.

The findings highlight the need for future interventions to focus on general practitioners and their practice. They are the key stakeholders in the care of autistic adults and managing the health services autistic adults access and receive.^[10,35,37–40,43,45–47]^ Future interventions could be focused on the importance of interdisciplinary care approaches for autistic adult healthcare, the access and delivery of healthcare from a general practitioner level, and the physical environment could be altered to better support patients with autism. General practice clinicians, hospital services including the emergency department, allied health, and specialist services play a vital role in an autistic person's life and, patient-provider level and service level factors can impact appropriate healthcare access.^[43]^

Concentrating on the transition age of autistic adults is vital, this is an important time in their life and their families lives that need to be considered. The services they stop accessing and the services they commence accessing, need to be well considered. Future interventions can focus on this area to support general practitioners’ resources and development of access to adult healthcare to better support autistic adult's health needs. The support coordination is vital and needs to be addressed.

A strength of this review is inclusion of autistic adult studies incorporating their views of the barriers of healthcare. The review considered multiple levels of healthcare that need further support to develop future interventions to better support this cohort. By establishing strict inclusion and exclusion criteria, a potential weakness of the study highlights that only 13 studies were eligible and accepted. Of these, none were Randomized Control Trials.

Consistent communication, sharing information, and building relationships is vital. Supporting the transition is important to build opportunities across agencies, which develops the reciprocal relationship required for successful transitions. In addition, Frye,^[42]^ found that acknowledging family's needs are important when addressing healthcare for autistic adults, as families may be involved in the health support coordination of their children. The research team were expecting to find literature related to how to enable participation in health services, however, we did not find any articles that were eligible for this systematic review. This is an important area of research, however, there is a paucity of evidence relating to the enablers of healthcare access and it is limited (Supplementary File 4- PRISMA-2009-Checklist-MS-Word).

## 5 Limitations

A weakness of the review was associated with the inclusion of both quantitative and qualitative research, which limited our ability to conduct a meta-analysis due to inconsistent results. Identifying specific enablers of healthcare was difficult to review because the included studies were diverse in participant numbers and demographics, measurement and analysis tools, quality and length of data collection. Such a range of studies led to limitations around a lack of consistency within the literature, the evidence-base was not coherent and there was no single variable measured amongst all articles, hence, it was not robust enough to perform a numeric meta-analysis or a sensitivity analysis.^[28]^

## 6 Conclusion

Autistic adults report many barriers specific to the delivery of healthcare but not particularly about the access to healthcare services. This systematic review highlights a global paucity of evidence for autistic adults’ access to healthcare. It is vital to support primary healthcare services to better enhance support for autistic adults as this is the first point of call for many individuals. A substantial number of autistic people are transitioning to adult healthcare and will need to access various services for overall wellbeing. Effective communication is the greatest barrier when accessing appropriate services and primary healthcare requires further support as general practitioners play a central role in liaising with services and finding appropriate support for autistic adults. This supports our initial hypothesis that poor communication and a lack of understanding about autistic needs are identified barriers. Therefore, interdisciplinary care approaches support transition and provides assistance when managing multiple chronic health conditions. Sharing medical information between clinicians and consistent communication between the patient, carers (if involved), and clinicians is vital in building relationships and transparency to provide better support for appropriate healthcare services and needs for the individual. This systematic review found that the transition from pediatric services to adult services requires a substantial number of clinicians and supports to support overall wellbeing. Similarly, past systematic reviews have focused on communication and found that communication was also a major barrier.^[26]^ Our systematic review has strengthened previous knowledge^[26]^ and supports the need for future interventions to focus on primary healthcare and effective communication for autistic adults. The implications for clinical practice remain part of the scope of practice for primary healthcare clinicians, however, future research can aim to create and implement evidence-based practices to better support access for this population. In conclusion, clinical guidelines on autistic healthcare access should be considered by all primary healthcare practices. Further empiric evidence is required to explore and understand the healthcare access barriers and enablers for autistic adults. This should include an analysis of various views and perceptions of people involved in the healthcare access of services for autistic adults.

## Author contributions

**Data curation:** Shenae Calleja.

**Analysis:** Shenae Calleja.

**Appraisal:** Shenae Calleja, Rachael McDonald, Amirul Islam and Jonathan Kingsley.

**Writing – original draft:** Shenae Calleja.

**Writing – review & editing:** Shenae Calleja, Rachael McDonald, Amirul Islam and Jonathan Kingsley.

## Supplementary Material

Supplemental Digital Content

## Supplementary Material

Supplemental Digital Content

## Supplementary Material

Supplemental Digital Content

## Supplementary Material

Supplemental Digital Content

## References

[1] Vogan V Lake JK Tint A Tracking health care service use and the experiences of adults with autism spectrum disorder without intellectual disability: a longitudinal study of service rates, barriers and satisfaction. Disabil Health J 2017;10:264–70. 27899267 10.1016/j.dhjo.2016.11.002

[2] Saqr Y Braun E Porter K Addressing medical needs of adolescents and adults with autism spectrum disorders in a primary care setting. Autism 2017;22:51–61. 28750547 10.1177/1362361317709970 PMC5788079

[3] American Psychiatric Association (APA). Diagnostic and Statistical Manual of Mental Disorders (DSM-5). Washington, DC: APA; 2013.

[4] Croen LA Zerbo O Qian Y The health status of adults on the autism spectrum. Autism 2015;19:814–23. 25911091 10.1177/1362361315577517

[5] Ayres M Parr JR Rodgers J A systematic review of quality of life of adults on the autism spectrum. Autism 2017;22:774–83. 28805071 10.1177/1362361317714988

[6] Fortuna RJ Robinson L Smith TH Health conditions and functional status in adults with autism: a cross-sectional evaluation. J Gen Intern Med 2016;31:77–84. 26361965 10.1007/s11606-015-3509-x PMC4700008

[7] Tyler CV Schramm SC Karafa M Chronic disease risks in young adults with autism spectrum disorder: forewarned is forearmed. Am J Intellect Dev Disabil 2011;116:371–80. 21905805 10.1352/1944-7558-116.5.371

[8] World Health Organisation. Autism Spectrum Disorders and Other Developmental Disorders: From Raising Awareness to Building Capacity; 2013. Available at: http://apps.who.int/iris/bitstream/10665/103312/1/9789241506618_eng.pdf. Accessed April 26, 2018.

[9] Hwang YI Srasuebkul P Foley KR Mortality and cause of death of Australians on the autism spectrum. Autism Res 2019;12:806–15. 30802364 10.1002/aur.2086

[10] Baldwin S Costley D The experiences and needs of female adults with high-functioning autism spectrum disorder. Autism 2016;20:483–95. 26111537 10.1177/1362361315590805

[11] Bishop KM Hogan M Janicki MP Guidelines for dementia-related health advocacy for adults with intellectual disability and dementia: National Task Group on Intellectual Disabilities and Dementia Practices. Intellect Dev Disabil 2015;53:2–9. 25633379 10.1352/1934-9556-53.1.2

[12] Hofvander B Delorme R Chaste P Psychiatric and psychosocial problems in adults with normal-intelligence autism spectrum disorders. BMC Psychiatry 2009;9:35. 19515234 10.1186/1471-244X-9-35 PMC2705351

[13] Smith KR Matson JL Behavior problems: differences among intellectually disabled adults with co-morbid autism spectrum disorders and epilepsy. Res Dev Disabil 2010;31:1062–9. 20452741 10.1016/j.ridd.2010.04.003

[14] Ali A Scior K Ratti V Discrimination and other barriers to accessing health care: perspectives of patients with mild and moderate intellectual disability and their carers. PLoS One 2013;8:e70855. 23951026 10.1371/journal.pone.0070855 PMC3741324

[15] Fitzpatrick SE Srivorakiat L Wink LK Aggression in autism spectrum disorder: presentation and treatment options. Neuropsychiatr Dis Treat 2016;12:1525. 27382295 10.2147/NDT.S84585 PMC4922773

[16] Kats D Payne L Parlier M Prevalence of selected clinical problems in older adults with autism and intellectual disability. J Neurodev Disord 2013;5:27. 24066979 10.1186/1866-1955-5-27 PMC3848965

[17] Orsmond GI Shattuck PT Cooper BP Social participation among young adults with an autism spectrum disorder. J Autism Dev Disord 2013;43:2710–9. 23615687 10.1007/s10803-013-1833-8 PMC3795788

[18] Kent R Carrington S Couteur A Diagnosing autism spectrum disorder: who will get a DSM-5 diagnosis? J Child Psychol Psychiatry 2013;54:1242–50. 23701321 10.1111/jcpp.12085 PMC4098079

[19] Pellicano E Dinsmore A Charman T What should autism research focus upon? Community views and priorities from the United Kingdom. Autism 2014;18:756–70. 24789871 10.1177/1362361314529627 PMC4230972

[20] Mukaetova-Ladinska E Perry E Baron M Ageing in people with autistic spectrum disorder. Int J Geriatr Psychiatry 2012;27:109–18. 21538534 10.1002/gps.2711

[21] McPheeters ML Warren Z Sathe N A systematic review of medical treatments for children with autism spectrum disorders. Pediatrics 2011;127:e1312–21. 21464191 10.1542/peds.2011-0427

[22] Warren Z McPheeters ML Sathe N A systematic review of early intensive intervention for autism spectrum disorders. Pediatrics 2011;127:e1303–11. 21464190 10.1542/peds.2011-0426

[23] Williams JG Higgins JP Brayne CE Systematic review of prevalence studies of autism spectrum disorders. Arch Dis Child 2006;91:8–15. 15863467 10.1136/adc.2004.062083 PMC2083083

[24] Mills E Jadad AR Ross C Systematic review of qualitative studies exploring parental beliefs and attitudes toward childhood vaccination identifies common barriers to vaccination. J Clin Epidemiol 2005;58:1081–8. 16223649 10.1016/j.jclinepi.2005.09.002

[25] Tregnago MK Cheak-Zamora NC Systematic review of disparities in health care for individuals with autism spectrum disorders in the United States. Res Autism Spectr Disord 2012;6:1023–31.

[26] Mason D Ingham B Urbanowicz A A systematic review of what barriers and facilitators prevent and enable physical healthcare services access for autistic adults. J Autism Dev Disord 2019;49:3387–400. 31124030 10.1007/s10803-019-04049-2 PMC6647496

[27] Beller EM Chen JK-H Wang UL-H Are systematic reviews up-to-date at the time of publication? Syst Rev 2013;2:36–136. 23714302 10.1186/2046-4053-2-36 PMC3674908

[28] Calleja S Islam FMA Kingsley J The disparities of healthcare access for adults with autism spectrum disorder: protocol for a systematic review. Medicine (Baltimore) 2019;98:e14480. 30762771 10.1097/MD.0000000000014480 PMC6408059

[29] Analytics C. EndNote X8; 2018. Available at: https://endnote.com. Accessed November 3, 2018.

[30] Pluye P, Robert E, Cargo M, et al. MMAT mixed methods appraisal tool; 2011. Available at: http://mixedmethodsappraisaltoolpublic.pbworks.com/w/file/fetch/84371689/MMAT%202011%20criteria%20and%20tutorial%202011-06-29updated2014.08.21.pdf. Accessed December 10, 2018.

[31] Lisy K Porritt K Narrative synthesis: considerations and challenges. Int J Evid Based Healthc 2016;14:201.

[32] Higgins J Sterne J Savović J Chandler J McKenzie J Boutron I Welch V A revised tool for assessing risk of bias in randomized trials. Cochrane Methods. Cochrane Database of Systematic Reviews 2016 10.

[33] Moher D, Liberati A, Tetzlaff J, Altman DG. The PRISMA flow diagram; 2009. Available at: http://prisma-statement.org/PRISMAStatement/FlowDiagram. Accessed December 10, 2018.

[34] Weiss JA Wingsiong A Lunsky Y Defining crisis in families of individuals with autism spectrum disorders. Autism 2013;18:985–95. 24254639 10.1177/1362361313508024 PMC4230960

[35] Nicolaidis C Raymaker D McDonald K Comparison of healthcare experiences in autistic and non-autistic adults: a cross-sectional online survey facilitated by an academic-community partnership. J Gen Intern Med 2013;28:761–9. 23179969 10.1007/s11606-012-2262-7 PMC3663938

[36] Baldwin S Costley D The experiences and needs of female adults with high-functioning autism spectrum disorder. Autism 2015;20:483–95. 26111537 10.1177/1362361315590805

[37] Tint A Weiss JA A qualitative study of the service experiences of women with autism spectrum disorder. Autism 2017;22:928–37. 28914071 10.1177/1362361317702561

[38] Foley K-R Pollack AJ Britt HC General practice encounters for young patients with autism spectrum disorder in Australia. Autism 2017;22:784–93. 28683578 10.1177/1362361317702560

[39] Rada RE Treatment needs and adverse events related to dental treatment under general anesthesia for individuals with autism. Intellect Dev Disabil 2013;51:246–52. 23909586 10.1352/1934-9556-51.4.246

[40] Hare DJ Pratt C Burton M The health and social care needs of family carers supporting adults with autistic spectrum disorders. Autism 2004;8:425–44. 15556960 10.1177/1362361304047225

[41] Havlicek J Bilaver L Beldon M Barriers and facilitators of the transition to adulthood for foster youth with autism spectrum disorder: perspectives of service providers in Illinois. Child Youth Serv Rev 2016;60:119–28.

[42] Frye L Fathers’ experience with autism spectrum disorder: nursing implications. J Pediatr Health Care 2016;30:453–63. 26700165 10.1016/j.pedhc.2015.10.012

[43] Nicolaidis Raymaker Ashkenazy “Respect the way I need to communicate with you”: healthcare experiences of adults on the autism spectrum. Autism 2015;19:824–31. 25882392 10.1177/1362361315576221 PMC4841263

[44] Nicolaidis C Kripke CC Raymaker D Primary care for adults on the autism spectrum. Med Clin North Am 2014;98:1169–91. 25134878 10.1016/j.mcna.2014.06.011 PMC4851469

[45] Dern S Sappok T Barriers to healthcare for people on the autism spectrum. Adv Autism 2016;2:2–11.

[46] Griffith GM Totsika V Nash S ‘I just don’t fit anywhere’: support experiences and future support needs of individuals with Asperger syndrome in middle adulthood. Autism 2011;16:532–46. 21610188 10.1177/1362361311405223

[47] Rogers CL Goddard L Hill EL Experiences of diagnosing autism spectrum disorder: a survey of professionals in the United Kingdom. Autism 2015;20:820–31. 26681687 10.1177/1362361315611109

