# Supplementary material for: Healthcare access for autistic adults: A systematic review
Source: Medicine (Baltimore). 2020 Jul 17;99(29):e20899. doi: 10.1097/MD.0000000000020899 (PMC7373620; doi:10.1097/MD.0000000000020899)
Supplement: Supplemental Digital Content [file medi-99-e20899-s001.docx]

Supplementary file 1

Key terms for database search

Full search strategy

| Search | Query |
| --- | --- |
| #1 | “(autism spectrum disorder* OR autism OR autistic OR ASD OR pervasive developmental disorder* OR Asperger OR neurodevelopmental disorder)” |
| #2 | “(Healthcare OR Health Services OR Health Care OR Health Management OR Hospital OR Medical OR Health Maintenance)” |
| #3 | “(Barrier OR Boundary OR Challenge)” |
| #4 | “(Enable* OR Facilitat*)” |

| Concept 1 | Concept 2 | Concept 3 | Concept 4 |
| --- | --- | --- | --- |
| Autism spectrum disorder | Healthcare | Barriers | Enablers |
