# Supplementary material for: Healthcare access for autistic adults: A systematic review
Source: Medicine (Baltimore). 2020 Jul 17;99(29):e20899. doi: 10.1097/MD.0000000000020899 (PMC7373620; doi:10.1097/MD.0000000000020899)
Supplement: Supplemental Digital Content [file medi-99-e20899-s002.docx]

Supplementary file 2: Sample elements for data extraction

| Sample elements for data extraction | | | | | | | | |
| --- | --- | --- | --- | --- | --- | --- | --- | --- |
| Number of participants | Source of participants | Participant demographics | Geographic location | Type of study | | Other co-conditions included | Reported outcomes | Level of healthcare identified that may need further support |
| Total sample size | Primary/secondary/tertiary healthcare  ASD organisations  Other relevant sources | Age  Gender  Socio-economic status  Level of education |  | |  | Intellectual disability  Other co-conditions | Quantitative: statistical significance  Qualitative: Category- or theme-level evidence  Mixed methods:  Statistical significance and category- or theme-level evidence |  |
